# Supplementary material for: Microglia and Perivascular Macrophages Act as Antigen Presenting Cells to Promote CD8 T Cell Infiltration of the Brain
Source: Front Immunol. 2021 Aug 30;12:726421. doi: 10.3389/fimmu.2021.726421 (PMC8435747; doi:10.3389/fimmu.2021.726421)
Supplement: Supplementary file 1 [file DataSheet_1.pdf]

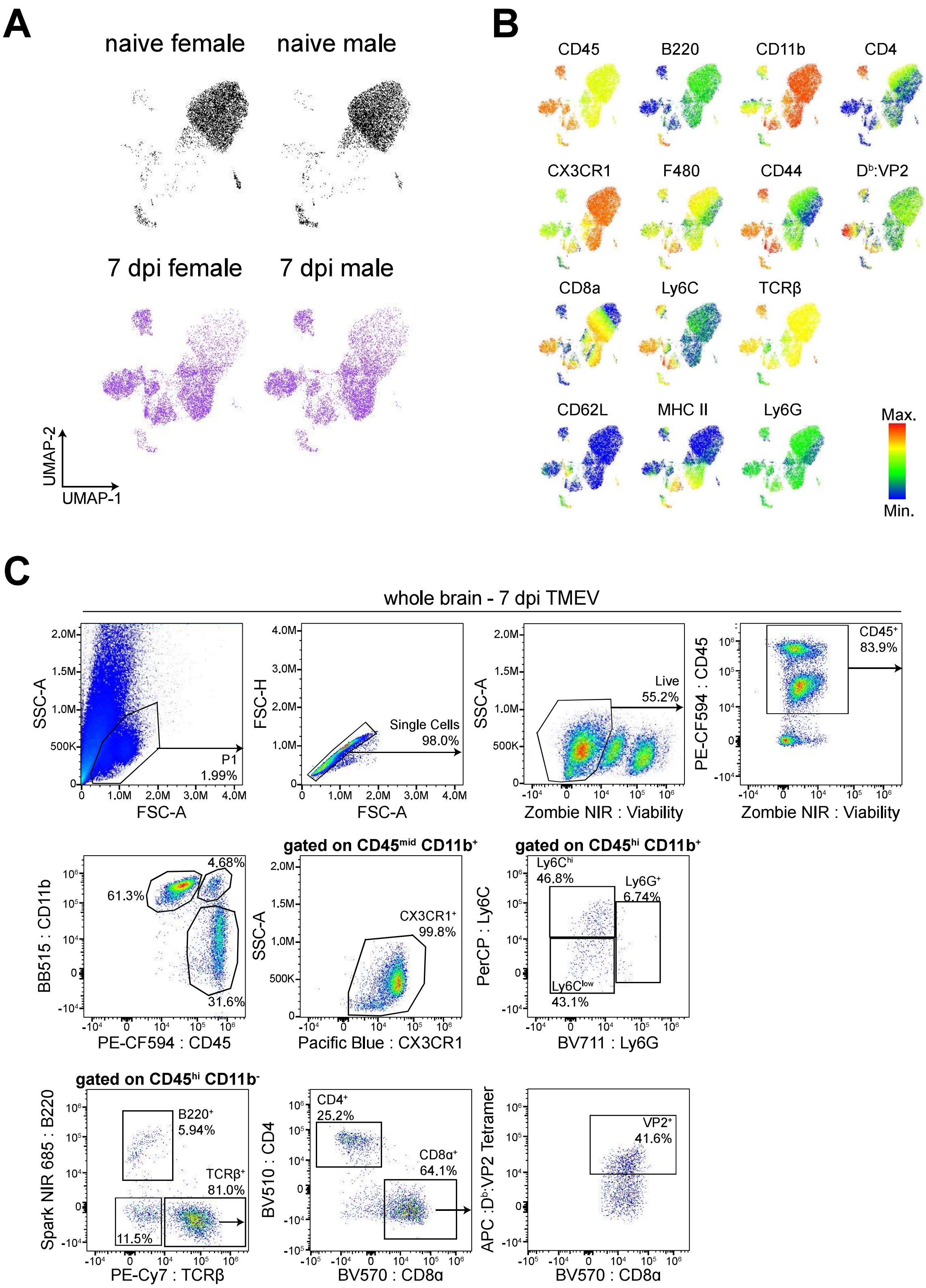
A
naive female
naive male
7 dpi female
7 dpi male
UMAP-2
UMAP-1
B
CD45
B220
CD11b
CD4
CX3CR1
F480
CD44
D^b:VP2
CD8a
Ly6C
TCRβ
CD62L
MHC II
Ly6G
Max.
Min.
C
whole brain - 7 dpi TMEV
SSC-A
FSC-A
P1
1.99%
FSC-H
Single Cells
98.0%
Live
55.2%
Zombie NIR : Viability
PE-CF594 : CD45
CD45+
83.9%
BB515 : CD11b
61.3%
4.68%
31.6%
gated on CD45mid CD11b+
CX3CR1+
99.8%
Pacific Blue : CX3CR1
gated on CD45hi CD11b+
Ly6Chi
46.8%
Ly6G+
6.74%
Ly6Clow
43.1%
PerCP : Ly6C
BV711 : Ly6G
gated on CD45hi CD11b-
Spark NIR 685 : B220
B220+
5.94%
TCRβ+
81.0%
11.5%
PE-Cy7 : TCRβ
BV510 : CD4
CD4+
25.2%
CD8α+
64.1%
BV570 : CD8α
APC :D^b:VP2 Tetramer
VP2+
41.6%

Figure S1. Analysis of immune compartment in naïve and TMEV-infected mice. Related to Figure 1. (**A**) UMAP visualization of the $CD45^+$ cell subsets isolated from the brain in Figure 1, color-coded by infection status and biological sex. (**B**) UMAP visualization of the $CD45^+$ cell subsets isolated from the brain, colored by expression of phenotypic cell surface markers used in flow cytometry analysis. (**C**) Representative flow cytometry gating strategies for manual analysis of immune cells in whole brain samples. Plots are representative of a TMEV infected mouse for ease of viewing. Data are representative of $\geq$ 3 independent experiments.

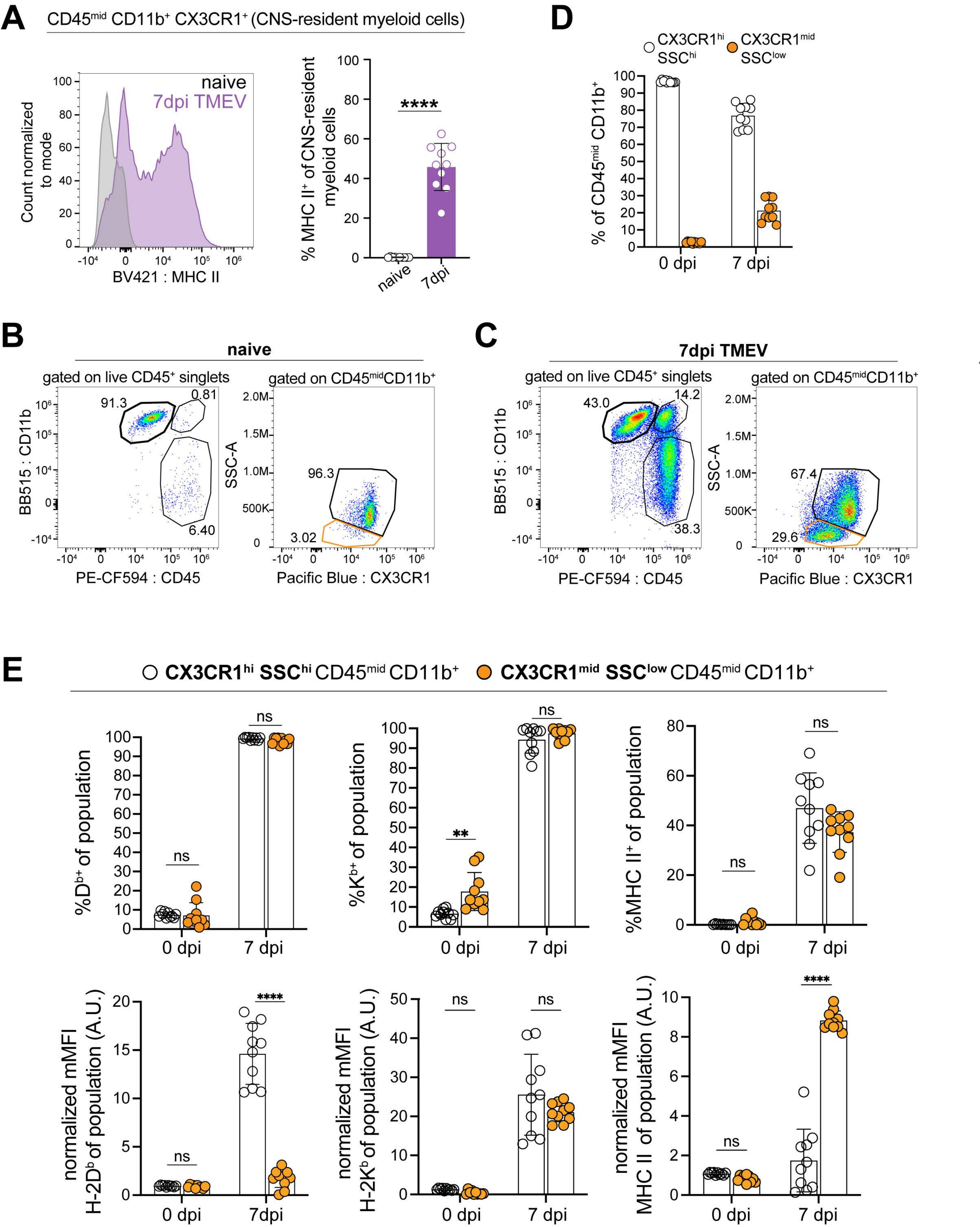

A
CD45mid CD11b+ CX3CR1+ (CNS-resident myeloid cells)
naive
7dpi TMEV
Count normalized to mode
BV421 : MHC II
% MHC II+ of CNS-resident myeloid cells
****
naive
7dpi
D
CX3CR1hi SSChi
CX3CR1mid SSClow
% of CD45mid CD11b+
0 dpi
7 dpi
B
naive
gated on live CD45+ singlets
gated on CD45midCD11b+
91.3
0.81
6.40
96.3
3.02
BB515 : CD11b
PE-CF594 : CD45
SSC-A
Pacific Blue : CX3CR1
C
7dpi TMEV
gated on live CD45+ singlets
gated on CD45midCD11b+
43.0
14.2
38.3
67.4
29.6
BB515 : CD11b
PE-CF594 : CD45
SSC-A
Pacific Blue : CX3CR1
E
CX3CR1hi SSChi CD45mid CD11b+
CX3CR1mid SSClow CD45mid CD11b+
%Db+ of population
%Kb+ of population
%MHC II+ of population
ns
**
normalized mMFI H-2Db of population (A.U.)
normalized mMFI H-2Kb of population (A.U.)
normalized mMFI MHC II of population (A.U.)
****
0 dpi
7 dpi
7dpi

Fig. S2. TMEV infection results in activation of CNS-resident myeloid cells and expression of MHC class I/II. Related to Figure 2. (**A**) MHC class II expression on CNS-myeloid cells during naïve and steady state conditions, demonstrated by representative histogram. The frequency of MHC II positive cells and normalized MFI of MHC II on the population is quantified (**B**) Representative flow cytometry plots of CNS-myeloid cells ($CD45^{mid}$ $CD11b^{+}$ $CX3CR1^{+}$) in the naïve state. (**C**) Representative flow cytometry plots of CNS-myeloid cells ($CD45^{mid}$ $CD11b^{+}$ $CX3CR1^{+}$) at 7 days post TMEV infection. (**D**) Quantification of $CX3CR1^{hi}$ SSC^{hi} and $CX3CR1^{mid}$ SSC^{mid} CNS-myeloid cells during naïve and TMEV infected conditions. (**E**) Expression of H-2Db, H-2Kb, and MHC II on $CX3CR1^{hi}$ SSC^{hi} (granular) and $CX3CR1^{mid}$ SSC^{mid} is quantified by percent-positive of population and normalized median fluorescence intensity. Data are representative of ≥ 3 independent experiments and presented as mean +/- SD, one-way ANOVA with Tukey's correction was used to assess statistical significance with ns $p \geq 0.05$. dpi, days post injury.

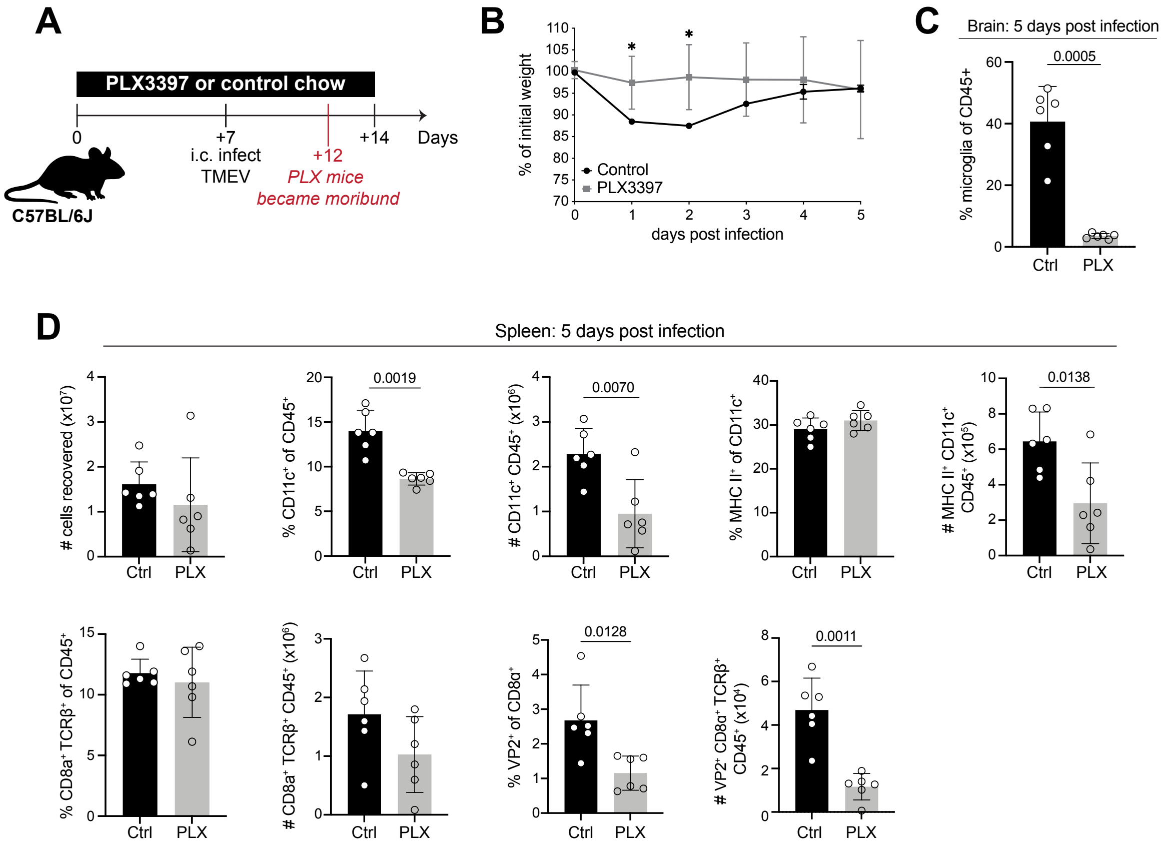

Fig. S3. Use of a CSF1R inhibitor reduces peripheral myeloid populations responsible for T cell priming. (**A**) Experimental timeline. C57BL/6 female mice were fed chow containing PLX3397 or control chow for 7 days prior to infection with TMEV and were continuously fed PLX throughout the course of infection. Flow cytometry analysis was executed when infected PLX treated mice became moribund at 5 dpi TMEV. (**B**) Weight loss as a result of infection was measured in control and PLX3397 treated mice. (**C**) The frequency of CNS-myeloid cells in the brain was quantified using flow cytometry in control and PLX3397 treated animals. (**D**) Analysis of splenic immune populations at 5 dpi, including: total cell number, frequency and number of CD11c^{+} cells, frequency and number of MHC II^{+} CD11c^{+} cells, frequency and number of CD8 T cells, and frequency and number of virus specific (D^{b}:VP2^{+}) CD8 T cells. Data are presented as mean +/- SD, significance was assessed using 2-tailed unpaired Student's *t* test with ns $p \geq 0.05$.

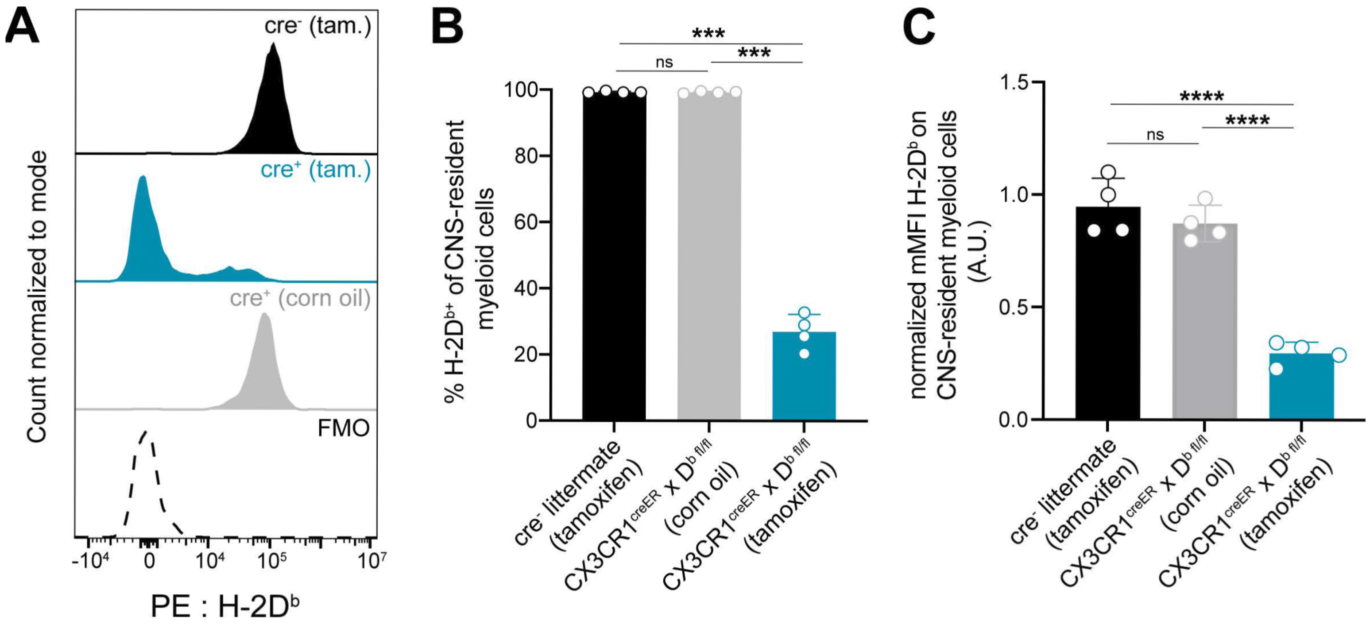

Fig. S4. No spontaneous recombination of floxed H-2D^{b} transgene occurs in CX3CR1^{cre}/D^{b} mice. Related to Figure 3. CX3CR1^{cre}/D^{b} animals were treated with vehicle control (corn oil) and compared to tamoxifen-treated cre^{+} and cre^{-} animals. (**A**) Representative histogram of H-2D^{b} expression on CNS-myeloid cells. (**B**) Quantification of percent D^{b} positive CNS-myeloid cells at 7 dpi TMEV (**C**) Normalized MFI of H-2D^{b} on CNS-myeloid cells. Data are shown as individual mice with mean from one independent experiment (n=4) of ≥ 2 experimental replicates. Error bars represent standard deviation. One-way ANOVA with Tukey's correction was used to assess statistical significance with ns $p \geq 0.05$.

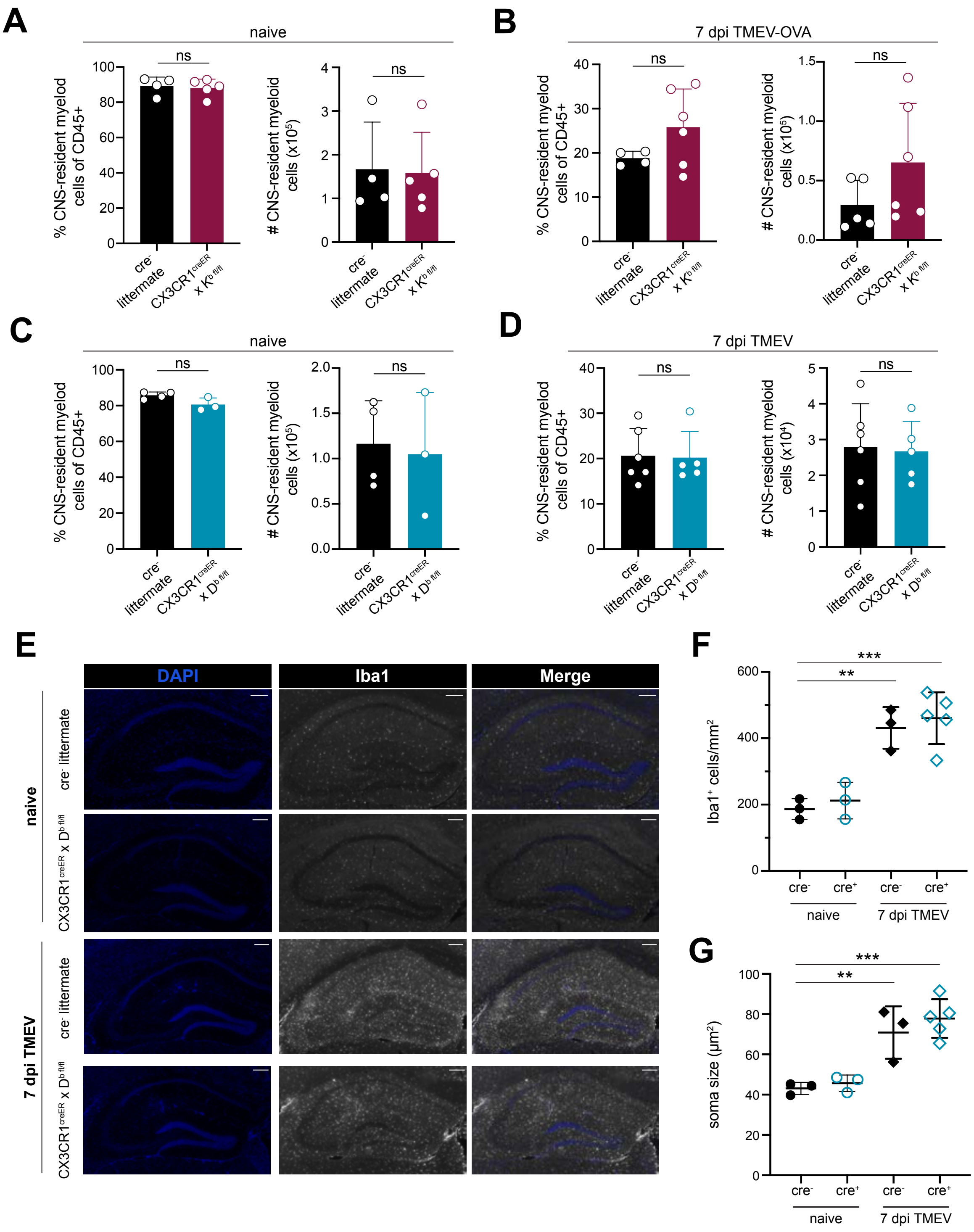
A
naive
ns
% CNS-resident myelod cells of CD45+
# CNS-resident myeloid cells (x10⁵)
cre⁻ littermate
CX3CR1creER x Kb fl/fl
B
7 dpi TMEV-OVA
C
naive
CX3CR1creER x Db fl/fl
D
7 dpi TMEV
cells (x10⁴)
E
DAPI
Iba1
Merge
naive
7 dpi TMEV
cre⁻ littermate
CX3CR1creER x Db fl/fl
F
Iba1⁺ cells/mm²
cre⁻
cre⁺
naive
7 dpi TMEV
**
***
G
soma size (µm²)

Fig. S5. No cell-intrinsic defects detected when CNS-myeloid cells lack MHC class I. Related to Figure 3. (**A-B**) Flow cytometic quantification of CNS-myeloid cells isolated from cre^- and $CX3CR1^{cre}/K^b$ mice during naïve conditions (**A**) and at 7 dpi with TMEV-OVA (**B**). (**C-D**) Flow cytometic quantification of CNS-myeloid cells isolated from cre^- and $CX3CR1^{cre}/D^b$ mice during naïve conditions (**C**) and at 7 dpi with TMEV (**D**). (**E**) Hippocampal sections collected at 0 and 7 days post TMEV infection were immunostained for Iba1 (white). Representative images are shown. (**F**) The density of hippocampal Iba1+ cells/mm^2 is quantified for the groups shown in (**E**). (**G**) The soma size of hippocampal Iba1+ cells were measured for the groups shown in (**E**). For flow cytometry experiments, CNS-myeloid cells are defined as live, single cells, expressing $CD45^{mid}CD11b^{+}CX3CR1^{+}$. Flow data are shown as individual mice with mean from one independent experiment (n=3-6) of at least three experimental replicates. Immunofluorescence data are shown as representative individual mice with mean from one independent experiment (n=3-5) of at least two experimental replicates. Iba1+ cell density is quantified from two hippocampal sections and plotted as average per mouse. Iba1+ cell soma size is quantified from 25 microglia per mouse and plotted as average per mouse. Error bars represent standard deviation. Two tailed Welch's *t* test or one-way ANOVA with Tukey's correction were used to assess statistical significance with ns $p \geq 0.05$.

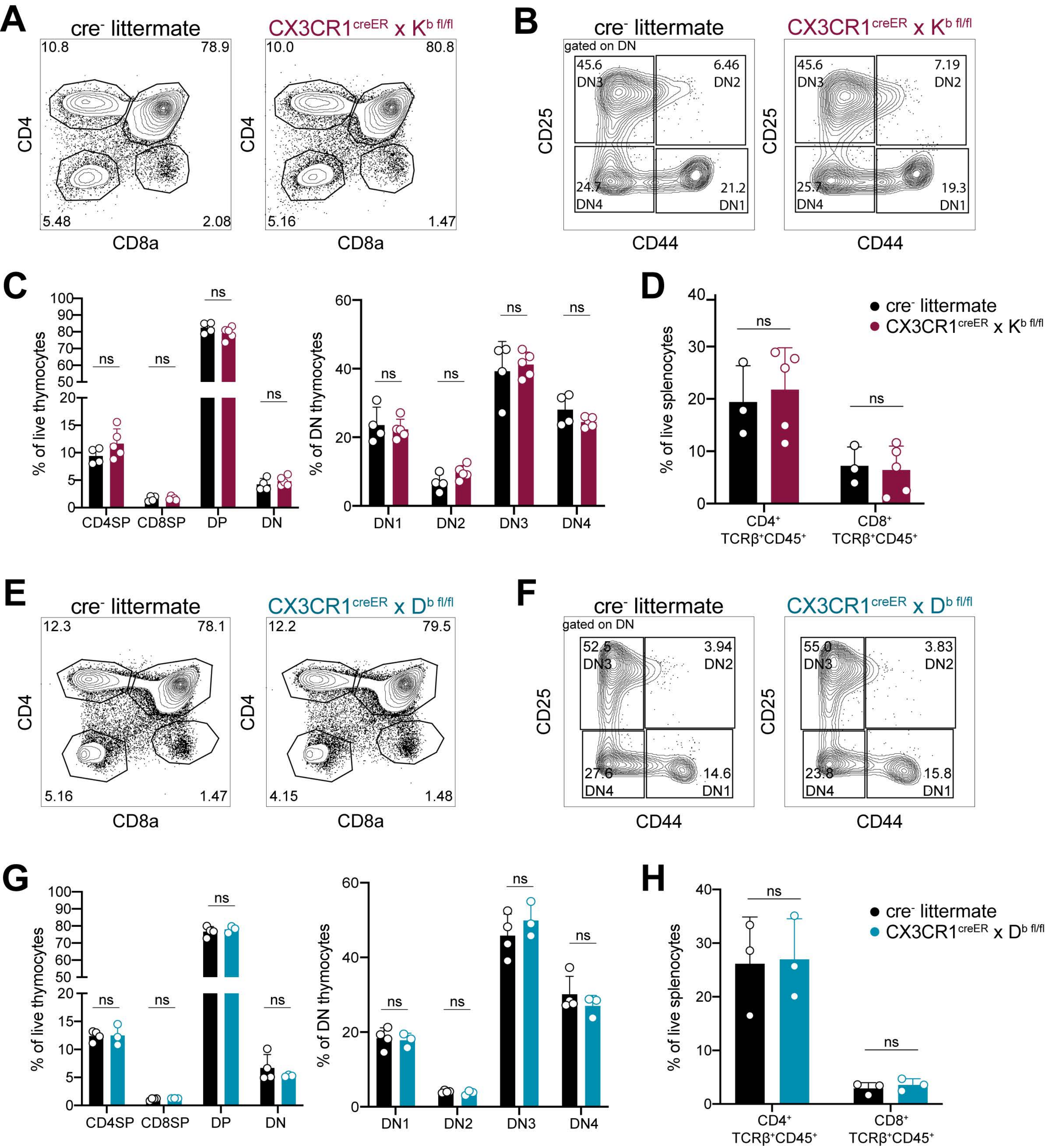
A
cre- littermate
CX3CR1creER x Kb fl/fl
10.8
78.9
5.48
2.08
10.0
80.8
5.16
1.47
CD4
CD8a
B
gated on DN
45.6 DN3
6.46 DN2
24.7 DN4
21.2 DN1
45.6 DN3
7.19 DN2
25.7 DN4
19.3 DN1
CD25
CD44
C
% of live thymocytes
CD4SP
CD8SP
DP
DN
% of DN thymocytes
DN1
DN2
DN3
DN4
ns
D
% of live splenocytes
CD4+ TCRβ+CD45+
CD8+ TCRβ+CD45+
cre- littermate
CX3CR1creER x Kb fl/fl
E
cre- littermate
CX3CR1creER x Db fl/fl
12.3
78.1
5.16
1.47
12.2
79.5
4.15
1.48
CD4
CD8a
F
gated on DN
52.5 DN3
3.94 DN2
27.6 DN4
14.6 DN1
55.0 DN3
3.83 DN2
23.8 DN4
15.8 DN1
CD25
CD44
G
% of live thymocytes
CD4SP
CD8SP
DP
DN
% of DN thymocytes
DN1
DN2
DN3
DN4
ns
H
% of live splenocytes
CD4+ TCRβ+CD45+
CD8+ TCRβ+CD45+
cre- littermate
CX3CR1creER x Db fl/fl

Fig. S6. Mice lacking MHC class I on CNS-myeloid cells have normal T cell development after tamoxifen treatment. Representative thymic gating strategies for tamoxifen treated cre^- littermates and $CX3CR1^{cre}/K^b$ are shown in (**A**) and (**B**). After gating on single, live, CD45+ cells, CD4 and CD8 were used to measure double negative (DN), double positive (DP), CD4 single positive (CD4SP), and CD8 single positive (CD8SP) (**A**). (**B**) Within the DN gate, DN1-4 were defined and quantified as follows: CD44+CD25- (DN1), CD44+CD25+ (DN2), CD44-CD25+ (DN3), and CD44-CD25- (DN4). (**C**) Frequencies of CD4SP, CD8SP, DP, DN, and DN1-4 are quantified between cre^- and $CX3CR1^{cre}/K^b$ animals. (**D**) CD4 and CD8 T cell frequencies were quantified in the spleens of cre^- and $CX3CR1^{cre}/K^b$ mice. (**E-H**) An identical analysis of T cell development was performed on cre^- littermates and $CX3CR1^{cre}/D^b$ animals. (**E-F**) Representative images and quantification of T cell subsets are shown. Data are represented as individual mice with mean from one independent experiment (n=3-5) out of at least three experimental replicates. Error bars represent standard deviation. Two tailed Welch's *t* test was used to assess statistical significance, with ns $p \geq 0.05$.

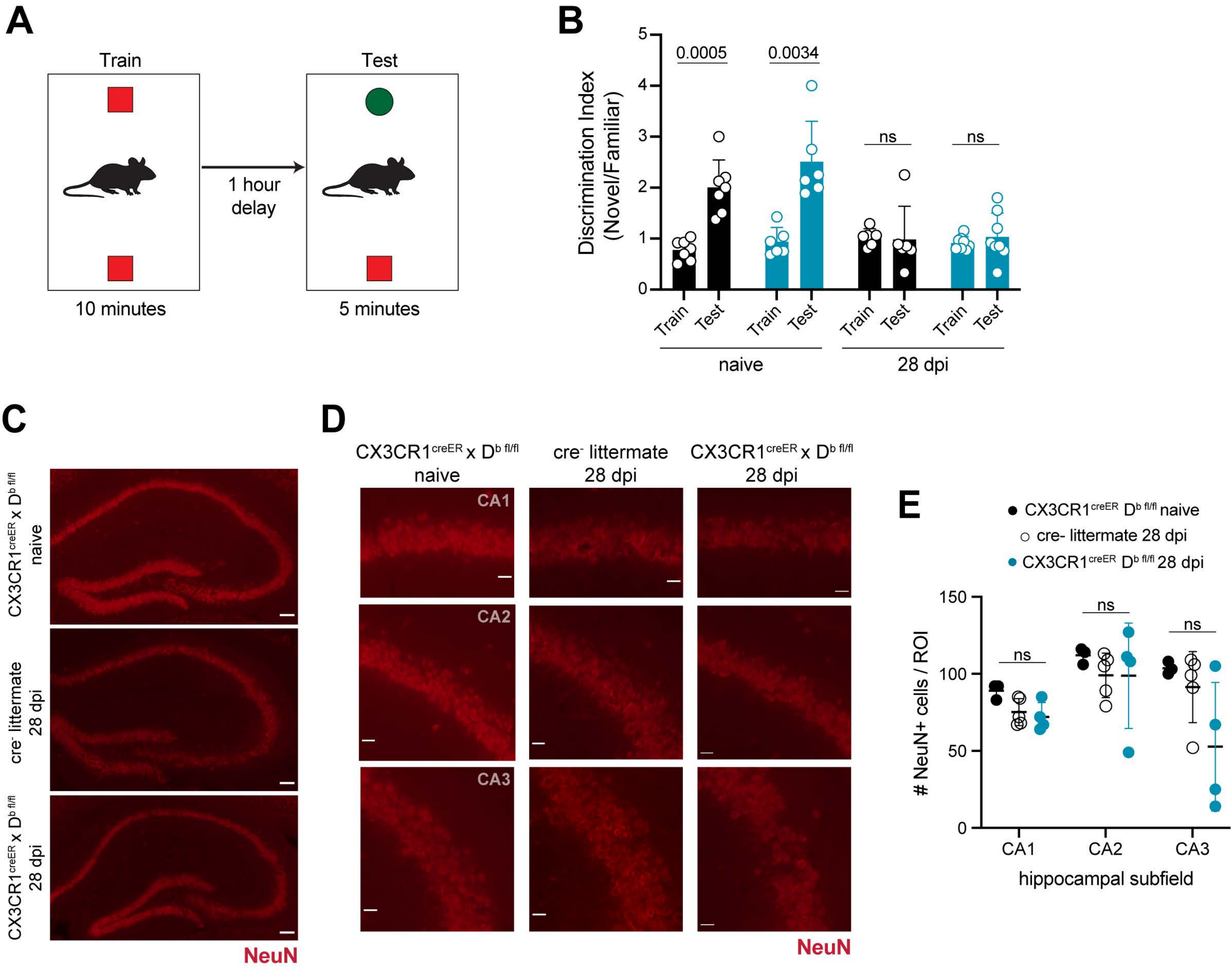
A
Train
Test
1 hour
delay
10 minutes
5 minutes
B
Discrimination Index
(Novel/Familiar)
0.0005
0.0034
ns
ns
Train
Test
naive
28 dpi
C
CX3CR1creER x Db fl/fl
naive
cre- littermate
28 dpi
CX3CR1creER x Db fl/fl
28 dpi
NeuN
D
CX3CR1creER x Db fl/fl
naive
cre- littermate
28 dpi
CX3CR1creER x Db fl/fl
28 dpi
CA1
CA2
CA3
NeuN
E
CX3CR1creER Db fl/fl naive
cre- littermate 28 dpi
CX3CR1creER Db fl/fl 28 dpi
NeuN+ cells / ROI
ns
hippocampal subfield

Fig. S7. Deletion of D^b from CNS-myeloid cells does not prevent cognitive deficits and damage to hippocampal neurons resulting from TMEV infection. Related to Figure 7. (**A**) Experimental paradigm for the Novel Object Recognition test of learning and recognition memory. After habituation, mice are exposed to two objects during the training period. After a delay, a novel object replaces a familiar object. Investigation of each object is quantified during both training and testing phases. (**B**) Discrimination index, or number of sniffs of novel object as compared to familiar object, in cre^- and $CX3CR1^{cre}/D^b$ mice under naïve conditions or after recovery from TMEV. (**C-D**) NeuN staining of the hippocampus in naïve and TMEV recovered control and $CX3CR1^{cre}/D^b$ animals. Equivalent ROIs in CA1, CA2, and CA3 were drawn and the number of NeuN+ cells were counted in each ROI. (**E**) Quantification of NeuN+ cells per ROI in each hippocampal area in recovered cre^- and $CX3CR1^{cre}/D^b$ mice compared to naive $CX3CR1^{cre}/D^b$ mice. Data are represented as individual mice with mean from one independent experiment (n=3-8) out of at least two experimental replicates. Error bars represent standard deviation. Two tailed Welch's *t* test or one-way ANOVA with Tukey's correction were used to assess statistical significance, with ns $p \geq 0.05$.
